# Translation, cultural adaptation and reproducibility of the Oxford Shoulder Score questionnaire for Brazil, among patients with rheumatoid arthritis: Tradução, adaptação cultural e reprodutibilidade do questionário Oxford Shoulder Score para o Brasil em pacientes com artrite reumatoide

**DOI:** 10.1590/1516-3180.2015.00800108

**Published:** 2015-12-08

**Authors:** Eider da Silva Lima, Jamil Natour, Emilia Moreira, Anamaria Jones

**Affiliations:** I PT, MSc. Doctoral Student, Rheumatology Division, Universidade Federal de São Paulo (Unifesp), São Paulo, Brazil.; II MD, PhD. Associate Professor, Rheumatology Division, Universidade Federal de São Paulo (Unifesp), São Paulo, Brazil.; III PT, PhD. Affiliated Professor, Rheumatology Division, Universidade Federal de São Paulo (Unifesp), São Paulo, Brazil.

**Keywords:** Translations, Validation studies, Quality of life, Shoulder pain, Questionnaires, Arthritis, rheumatoid

## Abstract

**CONTEXT AND OBJECTIVE:**

Although shoulder questionnaires validated for Brazil do exist, none of them are aimed at populations with rheumatic disease. We believe that the Oxford Shoulder Score (OSS) may be useful in this population. The objective of this study was to translate the OSS, adapt it to Brazilian culture and test its reproducibility.

**DESIGN AND SETTING:**

Validation study conducted in university outpatient clinics.

**METHODS:**

The OSS was translated into Portuguese by two English teachers and was then retranslated into English by two native English teachers. These translations were reviewed by a committee to establish the version of OSS-Brazil to be administered to 30 patients with rheumatoid arthritis (RA) and shoulder pain, in order to test the cultural adaptation. The validity and reproducibility was tested among another 30 patients with RA and shoulder pain, of both genders and aged 18 to 65 years. The internal consistency and reproducibility were analyzed. The following instruments were evaluated: OSS-Brazil; a numerical scale for shoulder pain; DASH; HAQ and SF-36.

**RESULTS:**

The internal consistency was 0.957 and the intra and inter-rater reproducibility was 0.917 and 0.861, respectively. A high level of correlation was found between OSS-Brazil and the following: HAQ (-0.663), DASH (-0.731) and the SF-36 domains of functional capacity (0.589), physical aspects (0.507), pain (0.624), general state of health (0.444), vitality (0.634) and mental health (0.578).

**CONCLUSION:**

OSS-Brazil was successfully translated and adapted, and this version exhibited good internal consistency, reliability and construct validity.

## RESUMO

**CONTEXTO E OBJETIVO:**

Apesar de existirem questionários de ombro validados para o Brasil, nenhum deles é voltado para a população com doenças reumáticas. Acreditamos que o Oxford Shoulder Score (OSS) possa ser útil nessa população. O objetivo deste estudo foi traduzir, adaptar à cultura brasileira e testar a reprodutibilidade do OSS.

**TIPO DE ESTUDO E LOCAL:**

Estudo de validação realizado nos ambulatórios da universidade.

**MÉTODOS:**

Foi realizada a tradução para o português por dois professores de inglês e depois a retradução para o inglês por dois professores nativos da língua inglesa. Essas traduções foram revisadas por um comitê para estabelecer a versão do OSS-Brasil, que foi aplicada em 30 pacientes com artrite reumatoide (AR) e dor no ombro para testar a adaptação cultural. A validade e a reprodutibilidade foram testadas em outros 30 pacientes com AR e dor no ombro de ambos os gêneros com idade entre 18 a 65 anos. Foram analisadas a consistência interna e a reprodutibilidade. Foram avaliados os seguintes instrumentos: OSS-Brasil; escala numérica de dor no ombro; DASH; HAQ e SF-36.

**RESULTADOS:**

A consistência interna foi 0,957 e a reprodutibilidade intra-avaliador e interavaliador foi 0,917 e 0,861, respectivamente. Foi observado alto nível de correlação do OSS-Brasil com HAQ (-0,663), DASH (-0,731) e os domínios do SF-36: capacidade funcional (0,589); aspectos físicos (0,507); dor (0,624); estado geral de saúde (0,444); vitalidade (0,634); saúde mental (0,578).

**CONCLUSÃO:**

O OSS-Brasil foi traduzido e adaptado com sucesso, com boa consistência interna, confiabilidade e validade de construção.

## INTRODUCTION

The impact of chronic diseases on individuals has led to development of quality-of-life instruments in order to better measure physical and mental factors, social wellbeing and factors that preserve function or control symptoms. Shoulder dysfunction is an important cause of mortality and incapacity,^1^ and is one of the most common peripheral complications in the general population.^2^^,^^3^ It is known that activities that use the arms or hands increase the risk of development of shoulder pain.^3^ It has been estimated that the incidence of shoulder problems ranges from 7 to 25 cases per 1,000 consultations with general practitioners.^4^ The prevalence of shoulder pain among adults under 70 years of age is between 7% and 27%, and it is between 13.2% and 26% for the over-70s.^5^

The Oxford Shoulder Score (OSS) is an instrument that specifically evaluates pain and quality of life in relation to inflammatory and degenerative shoulder diseases.^6^ It is a short, practical, reliable and valid questionnaire that is also sensitive to clinically important changes. However, it has not been adapted and validated for use among the Brazilian population. It may be important for detecting shoulder alterations and for measuring the impact of treatment.

Despite the existence of validated Portuguese-language shoulder questionnaires, none are directed towards the population with rheumatological diseases. The OSS was originally validated for several diseases including osteoarthritis and inflammatory arthritis.^6^ We believe the OSS may be very helpful in relation to clinical treatment of patients with rheumatological diseases.

Almost 12% of rheumatoid arthritis (RA) patients may have involvement of the shoulder joint, which can lead to pain and disability.^7^ No specific instrument for measuring shoulder abnormalities in RA patients currently exists.

## OBJECTIVE

The objective of this study was to translate the OSS, adapt it to Brazilian culture and test its reproducibility.

## METHODS

The study was performed in two stages: firstly, the original questionnaire was translated and adapted for Portuguese; and secondly, its reproducibility and external validity were tested in relation to the Brazilian population with rheumatoid arthritis and shoulder pain.

### Translation and adaptation process

The original authors of the OSS questionnaire authorized its use for cultural adaptation. The translation and cultural adaptation followed the standardization model proposed by Guillemin et al.^8^^,^^9^

The questionnaire was independently translated by two English teachers who provided two separate Portuguese translations. Both versions were presented to a committee of specialists consisting of a rheumatologist and two physiotherapists with experience in rheumatology rehabilitation. The committee analyzed the translations to check for errors, and then chose one version for each translated question, thereby creating an agreed consensus version.

After this process, the consensus version was back-translated into English by two native-speaker English teachers who were also proficient in Portuguese and had not had any contact with the original instrument. During this stage, the translators were not given any information about the study or the questionnaire.

The two English versions were then presented to the review committee and compared against the original questionnaire. This analysis showed that the original questionnaire and the consensus version were semantically equivalent, and the latter was then accepted as the final version. The final version of the questionnaire (Appendix 1) was used on 30 patients with RA and shoulder pain, in order to test its comprehensibility.

### Patients

Sixty patients with RA and shoulder pain complaints were selected consecutively at the Rheumatology Outpatient Clinic of the Federal University of São Paulo (Universidade Federal de Sao Paulo, UNIFESP): 30 in the first stage and 30 in the second stage. The study included male and female patients aged between 18 and 65 years who were classified as presenting RA under the criteria of the American College of Rheumatology (ACR),^10^ and who reported having shoulder pain within the last month.^11^ The study excluded individuals presenting any of the following: reduced shoulder range of motion due to skin lesions (e.g. burns), other autoimmune rheumatological diseases, neurological diseases, other upper-limb musculoskeletal diseases, shoulder trauma within the last week or shoulder instability; and also any patients who did not understand Portuguese.

This study was approved by the Ethics Committee of the UNIFESP, and all the patients who participated signed a written informed consent form.

### Evaluations

For the inter-observer reproducibility, the evaluations were performed during a single day by two physiotherapists and for the intra-observer reproducibility, the evaluations were carried out between 7 and 15 days after the initial evaluation, by the same physiotherapist.

### Evaluation instruments

- Oxford Shoulder Score - A questionnaire containing 12 questions for patients with inflammatory and degenerative shoulder diseases. It is not appropriate for patients with shoulder instability. Each question has 5 potential answers; carrying a score of between 0 and 4. The total score can range from 0 (worst) to 48 (best).^12^ The questions investigate pain and quality of life. The questionnaire is short, practical, reliable and valid, and is sensitive to clinically important changes.^6^
- Numerical Pain Scale (NPS) - A subjective evaluation of pain on a numerical scale of 0 to 10 centimeters, such that 0 represents absence of pain and 10 unbearable pain.^13^
- Disabilities of the Arm, Shoulder and Hand (DASH) scale - An instrument containing three modules: Module 1 (Q1) relates to sports and musical activities; Module 2 (Q2) relates to work; and Module 3 (Q3) relates to performance of activities. intensity of pain, symptoms of weakness, rigidity and paresthesia, negative effects on social activities, difficulty in sleeping and psychological harm, with reference to the previous week. Modules Q1 and Q2 each contain 4 questions, while Q3 is made up of 30 questions. Each question is scored using 5 scoring levels, and the final score of Q3 is calculated using the sum of the first 30 questions, from which the number of questions answered is subtracted and divided by 1.2; whereas for Q1 and Q2 the sum is subtracted by 4 and divided by 0.15.^14^
- Health Assessment Questionnaire (HAQ) - Evaluation of functional capacity: 20 questions split into 8 subscales relating to different aspects of activities within daily life in which the upper and lower limbs are used. The score is obtained by adding the highest scores from each subdivision and then dividing this partial result by 8. The scores range from 0 to 3, and the higher the resultant score is, the worse the individual’s functional capacity is.^15^
- Short Form-36 (SF-36) - Thirty-six questions divided into 8 domains that evaluate quality of life, covering factors relating to: functional capacity, physical limitations, pain, general state of health, vitality, social factors, emotional factors and mental health. The scores can range from 0 (worst) to 100 (best), and the higher the score is, the better the individual’s quality of life is.^16^
- The time taken to apply each questionnaire is noted.

### Statistical analysis

The sample was descriptively analyzed using means, standard deviations and percentages. Intraclass correlation coefficients were used to analyze inter and intra-observer reproducibility and the Spearman correlation test was used to ascertain the correlation between the OSS and the following: NPS scores for shoulder pain, DASH, HAQ and SF-36.

Cronbach’s alpha test was used to evaluate the internal consistency of the OSS. All questionnaire items were examined for correlations with the general score. Cronbach’s alpha test was also calculated through eliminating a single item from the total of 12 questions.^6^

All the data were analyzed using the SPSS software for Windows, version 17.0.

## RESULTS

A total of 221 patients classified as RA in accordance with the criteria of the American College of Rheumatology (2010), who reported having shoulder pain, were interviewed. Of these, 60 were included in the present study: 30 in the first stage and 30 in the second stage (Table 1). Most of the interviewees did not fulfill the inclusion criteria because they did not present shoulder pain or had juvenile idiopathic arthritis.

**Table 1.**
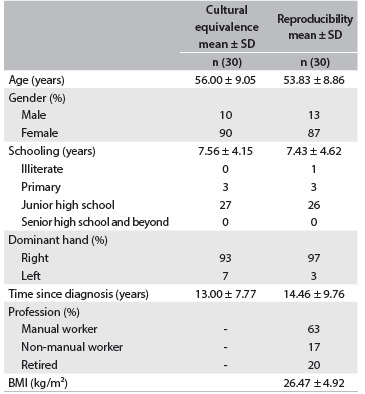
Demographic and clinical characteristics of the patients

Data are expressed as mean ± standard deviation (SD); BMI = body mass index.

For the cultural adaptation, the final version of the OSS was used among 30 patients in order to evaluate their comprehension of each question. Since the questions were understood by more than 90% of the patients interviewed, all the questions were retained without the need for any changes.

Regarding reproducibility, the patients answered all the questionnaires at the first evaluation and the times taken were recorded. Table 2 presents the absolute values of all the scores and the average times taken to apply the questionnaires. In relation to inter-observer reproducibility, no differences in the measurements made by the two evaluators were detected (P = 0.073) and the intraclass correlation coefficient (ICC) was 0.92, thus showing strong agreement between the evaluators. In relation to intra-observer reproducibility, no differences in the two measurements were detected (P = 0.290) and the intraclass correlation coefficient (ICC) was 0.86.

**Table 2.**
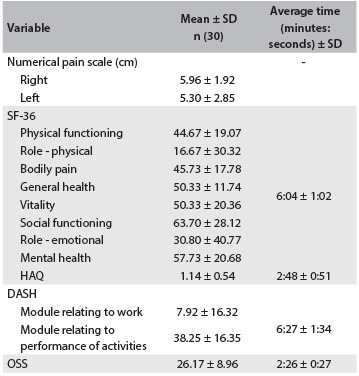
Scores and time taken for the instruments used

Data are expressed as mean ± standard deviation (SD); SF-36 = Medical Outcome Study Short Form-36 Health Survey; HAQ = Health Assessment Questionnaire; DASH = Disabilities of the Arm, Shoulder and Hand; OSS = Oxford Shoulder Score.

The internal consistency was high (Cronbach’s alpha = 0.93). Elimination of one answer item from each of the 12 questions in the questionnaire resulted in Cronbach’s alpha values of > 0.96. Item-total correlation analyses yielded coefficients greater than or equal to 0.52 in all cases (Table 3).

**Table 3.**
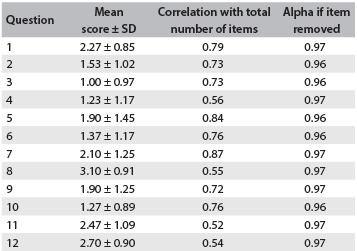
Oxford Shoulder Score internal consistency

SD = standard deviation.

Construct validity was tested using Spearman’s correlation coefficient (r = 0.75). Significant correlations between the Brazilian OSS and the HAQ, DASH and SF-36 questionnaires were observed, except in the domains of social factors and limitations due to emotional factors (Table 4).

**Table 4.**
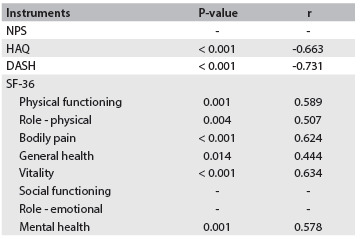
Correlations* between OSS and the NPS, HAQ, DASH and SF-36 domains

OSS = Oxford Shoulder Score; NPS = Numerical Pain Scale; HAQ = Health Assessment Questionnaire; DASH = Disabilities of the Arm, Shoulder and Hand; SF-36 = Medical Outcome Study Short Form-36 Health Survey; p > 0.05. *All correlations were analyzed using Spearman’s test. Spearman correlation r = -0.25 to 0 or 0 to 0.25 was considered to be small or null; r = -0.50 to -0.25 or > 0.25 to 0.50, weak correlation; r = -0.75 to -0.50 or > 0.50 to 0.75, moderate correlation; and r = -1.00 to -0.75 or > 0.75 to 1.00, strong correlation.

## DISCUSSION

The overall presence of RA in the general population ranges from 0.5% to 1% and approximately 11.5% of RA patients may have shoulder dysfunctions.^4^^,^^7^ Until now, no specific instrument was available in Brazil for evaluating the shoulders in RA cases. The instrument normally used for evaluating the upper limbs is DASH, which has been translated and validated for use among patients with RA. However, this instrument is generic and evaluates the entire upper limb,^14^ and is also lengthy and difficult to understand, as was observed in the current study. After making a comprehensive review of the literature, we chose the OSS for the translation and cultural adaptation process because it presents adequate validity and reproducibility and is also quick to apply, given that it is made up of easily comprehensible questions.^6^

Recently, a systematic review evaluated the development process, psychometric properties and administration of specific instruments for shoulder assessment. Its authors concluded that the OSS questionnaire should be the first choice for measuring function and disability among patients with shoulder dysfunction. Moreover, this instrument has shown high reproducibility, validity and responsiveness and is easy to administer, which makes it highly recommended.^17^ The OSS exists in several languages, including German, Italian, Dutch, Turkish, Korean and Danish.^18^^,^^19^^,^^20^^,^^21^^,^^22^^,^^23^

The importance of translating and validating a questionnaire for which the psychometric properties have already been tested comes from its unification of the language of clinical research. Through doing this, studies can be conducted around the world using the same evaluation instrument. This also facilitates comparison of data from different populations around the world.

Falcão et al. proposed a method for translation and cultural adaptation of instruments in order to reduce the time taken and cost of these studies.^23^ However, we chose to use Guillemin’s methodology, which is a specific method for translation and cultural adaptation of instruments. This methodology has been used in several studies and their criteria are internationally recognized.^8^^,^^9^

We chose to apply the questionnaire using interviewers because our patients’ low educational level would make self-administered questionnaires difficult to understand and answer. Our participants presented an average of 7.43 years of schooling, thus constituting a population of low educational level. This is common among patients using the Brazilian national health system (Sistema Único de Saúde, SUS), who generally have an average of 4 years of schooling, such that they know how to read and write.^24^

The original Oxford Shoulder Score presented a scoring range from 12 to 60 points. However, the questionnaire was recently revised and the scoring system changed such that the range is now from 0 to 48 points. Thus, this was the system of calculation that we used in our study.^6^^,^^12^

The average OSS scores obtained by interviewers I and II in the evaluations were similar. These results suggest that the functional involvement of these patients in relation to the shoulder was moderate, i.e. similar to the findings from other studies.^18^^,^^21^

In the present study, the intraclass correlation coefficient was 0.92 for intra-observer reproducibility. This figure is the same as the level in the original instrument, which was also 0.92. This shows that the questionnaire has good reproducibility.^6^

The inter-observer evaluation was performed after an interval of 7 to 15 days. This period was chosen because it was highly unlikely that the patient would remember the content of the questionnaire or that any substantive changes in their disease would have occurred during that time. Other studies have used shorter intervals of between 24 and 48 hours in order to avoid occurrences of changes to the participants’ state of health. Despite our use of a longer interval than those used in other studies, this did not affect the ICC result, as compared with the findings from other studies.^6^^,^^18^^,^^21^

According to the present study, the internal consistency was high, with Cronbach’s alpha of 0.93, which was similar to that of the original version, with a value of 0.87. In the correlations using the total number of items, Cronbach’s alpha was higher than 0.52 for all questions, thus showing a good level of differentiation. After elimination of one item from the total of 12 questions, the Cronbach’s alpha results were 0.96 or 0.97. These results were similar to those in the Italian version, which showed Cronbach’s alpha of 0.93, while the correlation using the total number of items was greater than 0.57.^21^

The correlation between the OSS and the DASH, HAQ and SF-36 questionnaires was moderate. This level of correlation was expected because DASH, HAQ and SF-36 also evaluate function and quality of life. The OSS had a positive correlation with SF-36, and a negative correlation with DASH and HAQ, i.e. it was inversely proportional, since higher score are better in DASH and HAQ.

The correlation between the OSS and the NPS was not significant, and this finding was similar to that of the original questionnaire because the purpose of the OSS is to measure quality of life and function. We expected to find a correlation between these variables, from the principle that with reductions in pain, there would be a positive response in relation to function and quality of life, although this is not always present in medical practice.

In relation to the average time taken to apply the questionnaires used, we noted that the OSS and HAQ took longer than the other questionnaires. However, HAQ evaluates overall function in patients with RA.

Within the study limitations, the greater prevalence of females in our sample may be explained by the greater prevalence of RA among females. However, we believe that this does not prevent the use of this instrument among male patients.^25^ The lack of a sensitivity test in the present study can also be considered to be a limitation, because this could detect changes as a result of interventions. This would make the instrument validation process more robust.

## CONCLUSION

The Oxford Shoulder Score questionnaire in Portuguese is a valid and reliable instrument for evaluating the quality of life and function of patients with rheumatoid arthritis and shoulder pain.
